# Severe Gastroparesis Leading to Hypoglycemia and Subsequent Seizures

**DOI:** 10.7759/cureus.30527

**Published:** 2022-10-20

**Authors:** Nardine Abdelsayed, Angel Juarez, Mary Carter

**Affiliations:** 1 Internal Medicine, Grand Strand Medical Center, Myrtle Beach, USA

**Keywords:** seizures, hypoglycemia, type 1 diabetes, hypoglycemia induced seizures, gastroparesis

## Abstract

Gastroparesis is a known complication in patients with diabetes mellitus (DM). This disorder has been known to make glycemic control difficult due to diabetic autonomic neuropathy, resulting in an increase in hypoglycemic episodes. On occasion, gastroparesis may be so severe that prokinetic medications and gastric pacemakers are not sufficient to control the symptoms, in which case some patients need to supplement their diet through a jejunal tube (J-tube) to bypass the stomach. Severe or long-lasting hypoglycemia may rarely be associated with epileptic seizures. We present a case of a 47-year-old female with a history significant for type 1 DM complicated by gastroparesis requiring a gastric pacemaker and J-tube placement who presented to the emergency department after having a witnessed seizure. Her glucose at that time was 27 mg/dl (normal 70-110 mg/dl) and she was treated appropriately with dextrose 25% solution and her glucose recovered to 110 mg/dl. Subsequently, seizure activity ceased.

## Introduction

Gastroparesis is defined as a delay in gastric emptying without an underlying obstruction. Roughly 25% of patients with gastroparesis have underlying diabetes mellitus as a major contributing factor. Prolonged hyperglycemia can lead to diabetic autonomic neuropathy (DAN) resulting in various manifestations such as tachycardia, orthostatic hypotension, erectile dysfunction, neurogenic bladder, and gastroparesis with labile glycemic control complicated by hypoglycemia. Patients with DAN also tend to have more hypoglycemic unawareness and hypoglycemic autonomic failure, which stuns the normal counter-regulatory response to hypoglycemia; subsequently, patients with DAN tend to have a greater risk of severe hypoglycemia. The pathogenesis of this counter-regulatory response is complex and attributable to multiple etiologies including a metabolic insult to nerve fibers, neurovascular insufficiency, autoimmune damage, and neurohormonal growth factor deficiency [1-12].

## Case presentation

Our patient is a 47-year-old female with a past medical history significant for hepatic sarcoidosis, type 1 diabetes mellitus complicated by severe gastroparesis (for which she had a gastric pacemaker implanted years prior and subsequently a jejunal tube placed three weeks prior) who presented after a witnessed seizure. On scene, her glucose level was 27 mg/dl and she was immediately given dextrose 25%. On arrival at our facility, the patient was more responsive, but still post-ictal and confused.

Further discussion with her husband revealed that, since the placement of the J-tube three weeks prior, they experienced difficulties with her tube feeds. The patient was undergoing five hours per day of tube feeds; however, her goal was 15 hours per day. The patient further reported that the feeds induced severe diarrhea and she was unable to tolerate higher portions. In addition, the patient was severely depressed, reporting a significant decrease in oral intake in addition to her tube feeds. She had drastically lost an estimated 30 lbs in three weeks. She also had numerous hypoglycemic episodes, as low as 40 mg/dl. She reported that she did not experience any symptoms of low glucose levels. She even decreased her usual long-acting insulin dosage with no change in the frequency of these events.

In the emergency department, her vitals showed tachycardia at 102 beats per minute (normal 60-100), and elevated blood pressure at 190/93 mmHg (normal <120/80), which improved to 166/88 mmHg without intervention. Initial labs are shown in Table 1.

**Table 1:** Initial Labs Obtained on Admission This table shows the general initial labs of the patient. The elevated alkaline phosphatase was noted to be chronic as the patient was previously diagnosed with hepatic sarcoidosis one year prior to admission.

	Lab value	Reference range	Interpretation
Hemoglobin	10.1 gm/dl	11.6-15.4	Decreased
Hematocrit	30.3%	34.9-44.1	Decreased
Mean corpuscular volume	91.5 fL	79.2-97.2	Normal
Lactic acid	8.78 mmol/l	<2	Increased
Aspartate aminotransferase	55 units/L	Normal, 10-56	Normal
Alanine aminotransferase	76 units/L	13-69	Increased
Gamma-glutamyl transferase	329 units/L	0-30	Increased
Bilirubin	1.0 mg/dL	0.1-1.1	Normal
Alkaline phosphatase	381 units.L	38-126 Units/L	Elevated

**Figure 1:**
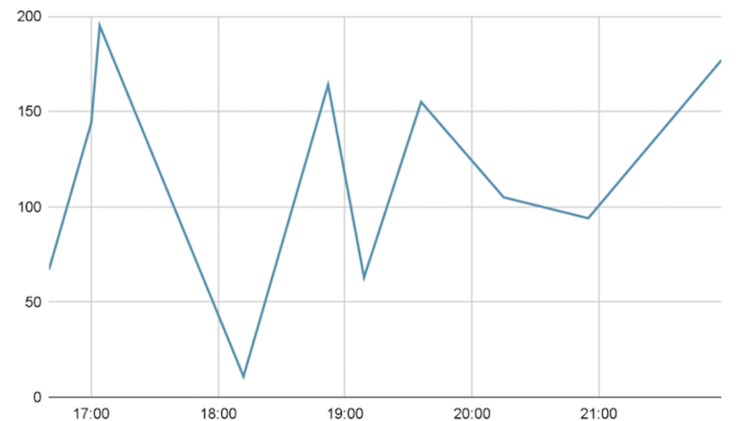
Glycemic Levels During First Four Hours of Hospitalization This is a graphical representation of the initial glucose levels of the patient. The glucose levels in mg/dl are on the y-axis, and time in hours is on the x-axis. A 50 mL dose of dextrose 50% IV injection was administered at 18:35 and repeated at 21:40.

During hospitalization, she continued to have significant hypoglycemic events, without any reported symptoms. She was then started on dextrose 10% at 50 mL per hour and her glucose levels improved. Her glucose levels during her first four hours of hospitalization are shown below in Figure 1. Notice that two hours after hospitalization, her glucose level dropped to 11 mg/dl. A dose of 50 mL dextrose 50% IV injection was given at 18:35 for a glucose level of 11 mg/dl and repeated at 21:40 for a glucose level of 63 mg/dl, and she was ultimately started on dextrose 10% at 50 mL per hour at 4:57 the following day for better glycemic control. Eventually, her tube feeds were changed to an alternative peptide-based formula, Vital, which she tolerated and was eventually back to her goal of 15 hours per day with improvement in glycemic control. The dextrose 10% was discontinued and her insulin regimen was slowly increased to optimize her glycemic control.

## Discussion

Gastroparesis is a known complication of DM. Other etiologies that lead to gastroparesis are hypothyroidism, autoimmune diseases, such as scleroderma, and nervous system disorders, such as Parkinson’s disease and multiple sclerosis. The most commonly known underlying cause of gastroparesis is DM, seen in 29% of individuals with the disease. Reports of gastroparesis vary based on the region and study design. One report found that 4.8% of patients with type 1 DM carried a diagnosis of gastroparesis [1], although that number is likely underestimated due to the disease being underdiagnosed. The symptomatology of gastroparesis includes postprandial fullness, nausea, vomiting, abdominal pain, bloating, and early satiety [2,8]. These symptoms can be debilitating in some cases causing malnourishment and severe hypoglycemia as seen in our patient.

Pathophysiology of gastroparesis in diabetes mellitus may be due to both vagus nerve dysfunction and changes in hormone secretion such as motilin and ghrelin. It is known that diabetic neuropathy is a complication of DM. Other possible causes include a metabolic insult to large nerve fibers, such as the vagus nerve, neurovascular insufficiency, autoimmune damage, and neurohormonal growth factor deficiency.

The vagus nerve is responsible for the regulation of a multitude of internal functions including digestion, cardiovascular regulation, vasomotor activity, and respiration. In digestion, the vagus nerve regulates ghrelin and motilin. Ghrelin is produced by endocrine cells in the stomach and it generally increases during periods of fasting and decreases after food intake, enhancing gastric emptying. Motilin is released in the duodenum during fasting and results in antral contraction. In individuals with gastroparesis, motilin, a prokinetic agent, has been noted to be low. For this reason, macrolides such as erythromycin may be used in the treatment of gastroparesis for their prokinetic properties [4]. Furthermore, these patients have been found to have lower stem cell factors (SCF) and Cajal growth factor cells, which results in deficits in the smooth muscle and "pacemaker" cells of the stomach. Eventually, there is increased stomach body relaxation, antral hypomotility, and atrium-duodenum-pylorus desynchrony [3]. With these findings, several changes occur that lead to abnormal duodenal motility and subsequent gastroparesis.

With changes in motility, postprandial emptying leads to effects in postprandial glucose levels, thus making glycemic control more difficult in patients with gastroparesis. It may also decrease patients' awareness of hypoglycemia by decreasing the typical symptoms such as dizziness, tachycardia, tremor, or diaphoresis [5]. In one study, 44 out of 54 patients reported more difficulty in controlling glucose levels following their diagnosis of gastroparesis. Its effect was found to be more pronounced in type 1 than in type 2 diabetics [2]. Another study found that hypoglycemia was reported in 25% of patients with gastroparesis as compared to 11% in patients with DM and no gastroparesis. As expected, this study also found that older age, lower socioeconomic status, longer duration of DM, and a higher hemoglobin A1c were factors that increased the risk of developing gastroparesis. It was interestingly found to be more prevalent in females than males (5.8% vs. 3.5%). It has also been shown that insulin pumps tend to be a protective factor against the development of gastroparesis [2].

Evaluation of gastroparesis begins with clinical recognition of the symptoms. Suspicion is elicited by symptoms of chronic nausea and vomiting, early satiety, abdominal pain, and postprandial fullness. Patient history will guide and exclude other differentials such as mechanical obstruction or malignancy. Upper GI endoscopy, abdominal CT, or MRI can assist in the diagnostic process. Once the mechanical obstruction is ruled out, gastroparesis is diagnosed by a gastric emptying study [1]. A radioisotope containing liquid or solid is used in the study, the study then quantifies gastric emptying as mild, moderate, or severe.

Treatment includes diet modifications consisting of smaller meals, a decrease in fiber and fat intake [3], optimizing glucose levels, and prokinetic agents such as metoclopramide or erythromycin. Antiemetics such as prochlorperazine and ondansetron can be used to provide symptomatic relief. Gastric pacemakers have also been used in refractory cases since 1970, which have shown an improvement in symptoms. Double-blind studies and numerous open-label studies have been conducted which have demonstrated improvement in patients with diabetic and idiopathic gastroparesis. However, it should be noted that not all open-label studies showed improvement with gastric electric stimulation (GES). It is recommended that physicians exercise caution with GES as a therapeutic strategy; clinical access shown in these studies must be weighed against the potentially deleterious effects of surgery [10]. Enteral feeding such as with a jejunostomy tube (J-tube) may also be necessary for some patients, such as our patient with persistent severe symptoms despite the above treatments [3].

Complications from gastroparesis vary based on severity, chronicity, and underlying etiologies. Some of these complications include hypoglycemia, severe protein-calorie malnourishment, esophageal injury from retching and vomiting, narcotic dependence, and iatrogenic procedure-related complications. Our patient suffered from severe hypoglycemia which resulted in a seizure. Severe hypoglycemic episodes in diabetics have been known to increase the risk of seizures. The mechanism of seizures in DM is multifactorial, including autoimmune influence (in type 1 DM) as well as large or rapid variations between hyper- and hypoglycemia. Hyperglycemia, specifically, may decrease the seizure threshold partially due to an increase in gamma-aminobutyric acid (GABA) metabolism and subsequent low levels [11]. Seizures occur due to the effect glucose has in relation to the excitability of neurons. Since the brain is dependent on glucose as its main fuel, abnormal levels, whether too high or low, disrupt neuronal activity putting patients with severe gastroparesis and frequent hypoglycemic episodes at higher risk of seizures.

One study found that only 1.2% of patients presenting with seizures are found to be hypoglycemic (defined in the study as glucose <60) [6], some of which may be an incidental finding. Another study following 156 patients in the intensive care unit (ICU) with a noted severe hypoglycemic episode (defined in the study as <45) demonstrated only one patient with resultant epileptic seizure [7].

Our patient had multiple factors that ultimately contributed to this epileptic event, including severe gastroparesis, long-acting insulin requirements in the setting of type 1 DM, depression with decreased oral intake as well as tube feed intolerance, and ultimately, severe hypoglycemia.

​​

## Conclusions

Our case highlights a rare consequence of severe gastroparesis in the setting of type 1 DM. Our patient developed severe hypoglycemia leading to seizures. Currently, gastroparesis continues to be underdiagnosed, leading to complications from the underlying etiology. If undiagnosed and untreated, complications can arise such as hypoglycemia, severe protein-calorie malnourishment, esophageal injury from retching and vomiting, narcotic dependence, and iatrogenic procedure-related complications. As in our patient, diabetic autonomic neuropathy provoked gastroparesis which led to a life-threatening complication of severe hypoglycemia and an epileptic event.
